# Gelastic epilepsy in combination with hypothalamic hamartoma and partial agenesis of the corpus callosum: A case report and review of the literature

**DOI:** 10.3892/etm.2013.1331

**Published:** 2013-10-08

**Authors:** BOCHAO CHENG, CHONGRAN SUN, SHIGUANG LI, QIYONG GONG, SU LUI

**Affiliations:** 1 Huaxi MR Research Center (HMRRC), Department of Radiology, West China Hospital of Sichuan University, Chengdu, Sichuan 610041, P.R. China; 2 Epilepsy Center, Department of Neurosurgery, The Second Affiliated Hospital, Zhejiang University School of Medicine, Hangzhou, Zhejiang 310009, P.R. China

**Keywords:** gelastic epilepsy, hypothalamic hamartoma, partial agenesis of the corpus callosum

## Abstract

Gelastic epilepsy has been reported to originate from various conditions, particularly from hypothalamic hamartoma (HH). In the present study, we report a patient with gelastic seizures (GSs), followed by complex partial and tonic-clonic seizures. Magnetic resonance imaging (MRI) revealed a rare combination of HH and partial agenesis of the corpus callosum (ACC). Following resectioning of the HH, the seizures were reduced, but not fully controlled, with medication by the one year follow-up. HH and partial ACC patients may experience seizures; the seizures in the case presented in this study may have originated from HH, partial ACC or both. Considering the fact that seizure frequency reduced following surgery, they may have mainly occurred from HH. Additionally it was considered to be likely that the seizures following surgery were due to secondary epileptogenesis, partial ACC, or both.

## Introduction

Gelastic seizures (GSs) are known as ‘laughing seizures’ as they may look like bouts of brief, unprovoked, uncontrollable laughter or giggling with a facial contraction. GS symptoms often start at an early age, occasionally even starting in infancy. GSs often have a high frequency, occurring several times daily. Gradually, GSs may grow worse with age, accompanied by ensuing of other seizure types and inevitably leading to cognitive and behavioral impediment. Although they are rarely reported, GS cases arising from temporal or frontal regions are commonly considered to be the typical manifestation of hypothalamic hamartoma (HH) (1). Most cases of gelastic seizures associated with the evolution of hypothalamic hamartoma reported resistant to drug therapy (2), thus an early excision of the hamartoma is required. Agenesis of the corpus callosum (ACC), an uncommon congenital cerebral malformation, often manifests as seizures and psychomotor retardation (3). The deficit may be complete or partial and is difficult to detect prior to magnetic resonance imaging (MRI) and it is usually observed in conjunction with other brain anomalies (3).

To the best of our knowledge, only HHs associated with complete ACC have been reported (4,5). No studies regarding partial ACC associated with HH have been reported with surgical outcomes, which may have different symptoms and underlying mechanisms compared with HHs associated with complete ACC. In this case report, we examined a rare case with a combination of HH and partial ACC, which manifested as GSs followed by complex partial and general seizures. We also reviewed the literature and discussed the mechanism underlining the symptoms and surgical outcomes.

## Case report

### Patient history

A 6-year-old right-handed male, had exhibited intermittent laughter and convulsions for >5 years. The patient was the first child of healthy parents with a non-consanguineous marriage, and had been born uneventfully by a normal vaginal delivery without any perinatal problems. There was no family history of epilepsy. The seizures started when the patient was 8 months old, and manifested as brief intermittent staring followed by uncontrollable laughter and giggling during the day and night. The seizures lasted for a few seconds and occurred 4–10 times per day and were initially controlled with lamotrigine (100 mg/day) and sodium valproate (750 mg/day).

### Examination

**Figure 1:**
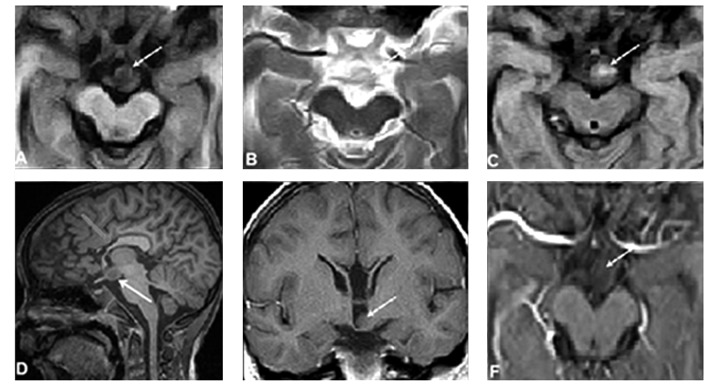
Pre-surgical 3.0 T magnetic resonance imaging (MRI) examination of the patient. (A-E) MRI revealed the ovoid-shaped hypothalamic hamartoma (HH) located at the the suprasellar region (indicated by arrow). (F) No enhancement of the HH MRI with intravenous, gadolinium-based contrast (indicated by arrow). (A) T1-weighted spin-echo (SE) axial slice. (B) T2-weighted SE axial slice. (C) Fluid attenuated inversion recovery (FLAIR) axial slice. (D) T1-weighted SE sagittal slice. The corpus callosum with absence of the rostrum, the genu and frontal part of the body of corpus callosum (indicated by arrow). (E) T1-weighted SE coronal sclices. (F) T1-weighted SE axial enhanced slice.

**Figure 2:**
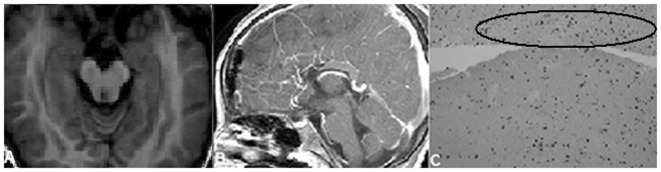
(A and B) Post-surgical 3.0 T magnetic resonance imaging (MRI) examination of the patient following the removal of the hypothalamic hamartoma (HH). (C) Hematoxylin and eosin-stained section examination of the surgical material. Disarrayed distribution of neurons with abnormal neurons and cluster of small neurons (indicated by black circle). Original magnification, ×400.

The first scalp electroencephalogram (EEG)did not reveal any abnormal findings. Following this, the frequency of seizures increased to 7–8 times per hour and gradually deteriorated to GSs followed by complex partial seizures and tonic-clonic seizures. When the patient was five years old he was admitted to the West China Hospital of Sichuan University (Chengdu, China). Neurological examination was conducted without observing any pathological features, and motor and cognitive development were found to be normal. Topiramate (100 mg/day), lamotrigine (100 mg/day) and sodium valproate (750 mg/day) were administered orally but failed to control the deterioration of the seizures. Video-EEG revealed that the epileptic spikes originated from the left hemisphere, mainly the frontal and temporal regions, followed by widespread bilateral epileptiform discharges in which the type of spike activity differed greatly between the right and left hemispheres. A contrast-enhanced cerebral MRI examination was performed and revealed a well-demarcated intrahypothalamic mass (Fig. 1A–F), which was ovoid-shaped and located in the suprasellar region. The mass was iso-intense to the grey matter and non-enhanced on the post-gadolinium images (Fig. 1F). Furthermore, the T1-weighted spin-echo (SE) (TR-450/TE-25) sagittal slice revealed signs of partial ACC (Fig. 1D). Since the progressive epilepsy and other symptoms may be ameliorated by surgical intervention, early surgical excision of the HH was suggested. Following surgical resectioning and lamina terminalis fenestration, the mass in the suprasellar region was completely removed (Fig. 2A and B). The mass was confirmed as HH by post-surgical histological examination (Fig. 2C).

### Follow-up

The patient was followed up at the outpatient department of West China hospital following surgery. Anti-seizure therapy with lamotrigine, 50mg/day, was provided following surgery. At the one year follow-up, the seizures had been reduced to 2–4 times/day.

## Discussion

HH is associated with a variety of neurological and/or endocrinal abnormalities. The usual symptoms of a patient with HH include GSs, precocious puberty and developmental retardation (6). HH may be classified into sessile (intrahypothalamic) and pedunculated (parahypothalamic). Sessile HH is often associated with epilepsy, while the pedunculated variety presents with precocious puberty (7). MRI reveals the HH, which often appears isointense to grey matter without contrast enhancement (Fig. 1).

The nodules, or clusters of small neurons, were considered to be the universal histological feature of HH lesions associated with pharmacologically refractory epilepsy (2). Even rare findings such as an independent epileptogenic focus outside of the hypothalamus were reported. The intrinsic epileptogenicity of the HH, particularly when the sessile HH protrudes into the third ventricle has been previously confirmed (8).

Generally, AEDs (Antiepileptic drugs) are ineffective for GSs associated with HH and rarely prevent cognitive and behavioral deterioration. In a long-term follow-up retrospect review (6 years on average), only 3 of 8 HH patients with GSs and other types of seizures achieved acceptable control by AEDs (9). Several studies have demonstrated that the resection of HH is effective for long-lasting control of seizures and may alleviate the behavioral and cognitive abnormalities (10,11). Thus, early surgical excision of the HH is recommended.

ACC is a cerebral malformation mainly caused by the abnormalities of chromosomes 8, 11, 13–15 and 18 from the cells of a fetus (12) and is often associated with other brain anomalies such as interhemispheric cysts with hydrocephalus, Dandy-Walker syndrome and neuronal migrational disorder (3). The corpus callosum functions as the anatomic connection for transferring information between hemispheres. The defect may be complete or partial, depending on the stage of callosal development inhibition (3). Although ACC is not lethal, patients with ACC may present with neurological problems, such as complex partial seizures, intellectual impairment and psychosis (3).

Several studies have postulated that the deterioration of seizures with EEG abnormalities may be a form of secondary epileptogenesis or a ‘kindling effect’ in the neocortex (13). Although oxcarbazepine, carbamazepine, topiramate, valproic acid and levetiracetam were frequently used to control GSs, previous studies on HH combined with complete ACC used conservative treatments and the number of general seizures decreased by 75% using anticonvulsant therapy (4,5). However, in this case we were unable to decrease the number of refractory seizures using anticonvulsant therapy which may be due to the differences between partial and complete ACC as complete ACC theoretically inhibits ictal electrical impulses transmitted to the other side of the brain. The removal of HH would be the treatment of choice in this case.

HH and ACC patients are capable of experiencing seizures, which in the present case may have originated from HH, partial ACC or both. Considering the fact that the frequency of the seizures was reduced following surgery, the seizures may have mostly occurred from HH. However, it was considered to be unlikely that the seizures following surgery were due to the secondary epileptogenesis or partial agenesis of the corpus callosum, or both. Furthermore, whether the co-occurrence of HH and ACC in our patient was a coincidence or a new syndrome required clarification. Clarifying this question requires further clinical evidence, long-term clinical follow-up and genetic studies, which are likely to be performed if a candidate gene is eventually identified.
